# Focal Seizures and Posterior Reversible Encephalopathy Syndrome as Presenting Signs of IgA Vasculitis/Henoch-Schoenlein Purpura—An Educative Case and Systematic Review of the Literature

**DOI:** 10.3389/fneur.2021.759386

**Published:** 2021-11-15

**Authors:** Dominik Funken, Friedrich Götz, Eva Bültmann, Imke Hennies, Janina Gburek-Augustat, Julya Hempel, Frank Dressler, Ulrich Baumann, Christian Klemann

**Affiliations:** ^1^Department of Pediatric Pneumology, Allergology and Neonatology, Hannover Medical School, Hanover, Germany; ^2^Institute of Diagnostic and Interventional Neuroradiology, Hannover Medical School, Hanover, Germany; ^3^Department of Pediatric Nephrology, Hepatology and Metabolic Disorders, Hannover Medical School, Hanover, Germany; ^4^Division of Neuropediatrics, Hospital for Children and Adolescents, University Hospital Leipzig, Leipzig, Germany

**Keywords:** Henoch-Schönlein purpura (HSP), CNS involvement, IgA vasculitis, cerebral vasculitis, small-vessel vasculitis, treatment, review, encephalopathy syndrome

## Abstract

**Background:** IgA vasculitis/Henoch-Schoenlein purpura (IgAV/HSP) is a systemic small vessel vasculitis of unknown pathogenesis predominantly affecting children. While skin, GI tract, joints, and kidneys are frequently affected and considered, central nervous system (CNS) involvement of this disease is underestimated.

**Methods:** We provide a case report and systematically review the literature on IgAV, collecting data on the spectrum of neurological manifestations.

**Results:** We report on a 7-year-old girl with IgAV who presented with diplopia and afebrile focal seizures, which preceded the onset of purpura. Cranial magnetic resonance imaging was consistent with posterior reversible encephalopathy syndrome (PRES), showing typical focal bilateral parietal swelling and cortical and subcortical high signal intensities on T2-fluid attenuated inversion recovery (FLAIR) images predominantly without diffusion restriction. Cerebrospinal fluid analysis and blood tests excluded systemic inflammation or vasculitis. Interestingly, hypertension was not a hallmark of the developing disease in the initial phase of PRES manifestation. Renal disease and other secondary causes for PRES were also excluded. Supportive- and steroid treatment resulted in restitution *ad integrum*. Reviewing the literature, we identified 28 other cases of IgAV with CNS involvement. Severe CNS involvement includes seizures, cerebral edema, or hemorrhage, as well as PRES. Thirteen patients fulfilled all diagnostic criteria of PRES. The mean age was 11.2 years (median 8.0, range 5-42 years), with no reported bias toward gender or ethnic background. Treatment regimens varied from watchful waiting to oral and intravenously steroids up to plasmapheresis. Three cases showed permanent CNS impairment.

**Conclusion:** Collectively, our data demonstrate that (I) severe CNS involvement such as PRES is an underappreciated feature of IgAV, (II) CNS symptoms may precede other features of IgAV, (III) PRES can occur in IgAV, and differentiation from CNS vasculitis is challenging, (IV) pathogenesis of PRES in the context of IgAV remains elusive, which hampers treatment decisions. We, therefore, conclude that clinical awareness and the collection of structured data are necessary to elucidate the pathophysiological connection of IgAV and PRES.

## Introduction

IgA vasculitis [IgAV, formerly known as Henoch-Schönlein purpura (HSP)] is an immune complex-mediated small-vessel vasculitis with an incidence of about 20 per 100,000 children per year, thus representing the most common vasculitis in children (1, 2). Half of the affected patients are below the age of six, and 90% are under 10 (3). However, IgAV can also occur in adults. Males are affected twice as often compared to female patients (2). The inflammation is characterized by the deposition of abnormal immune complexes in the wall of blood vessels. The exact pathogenesis is elusive, but clinical and experimental data point toward an infectious trigger and genetic susceptibility such as abnormalities in the IgA1-molecule. The vast majority of children present with non-thrombocytopenic purpura, arthralgia/arthritis, and abdominal pain. Additionally, multiple manifestations in other organs, such as the kidney (glomerulonephritis), intestine (intussusception), lungs (alveolar hemorrhage), and central nervous system (seizures, cerebrovascular thrombosis) have been reported (2, 4, 5). Posterior reversible encephalopathy syndrome (PRES) has been anecdotally reported in patients with IgAV (6–10).

PRES is clinically characterized by seizures, encephalopathy, headache, and visual disturbance (11, 12).

Although initially reported in patients with malignant hypertension, elevated blood pressure is not mandatory for the diagnosis of PRES (12). The diagnosis is based on clinical presentation, known risk factors, and vasogenic edema on magnetic resonance imaging (MRI) of the brain. Cortical or subcortical hyperintensities on T2-weighed images most commonly affect the parieto-occipital region. In the context of IgAV, it can be difficult to distinguish cerebral vasculitis from encephalopathy in the overlapping setting of PRES and hypertension (hypertensive encephalopathy; HE). Severe complications may accompany PRES, and neurological sequelae may persist in some patients if not expeditiously recognized (13). Here, we extend the clinical spectrum of CNS involvement of IgAV by reporting a girl with seizures and PRES as the initial manifestation of IgAV. This case is unique as the neurological manifestations preceded the typical clinical signs of IgAV and thus poses an educative diagnostic challenge. Reviewing the literature revealed that seizures and PRES appear to be an underestimated feature of IgAV with the potential risk of severe sequelae if not diagnosed and treated in time.

## Materials and Methods

Informed consent for collecting the patients' history and clinical data were obtained from the patient and her guardians. The study was performed in accordance with the declaration of Helsinki and the local institutional review board (Ethic Committee, Hannover Medical School, Hannover). We used the CARE checklist when writing our report (14).

A systematic literature review of the PubMed database consisting of the US National Library of Medicine and Excerpta Medica dataBASE was conducted according to the PRISMA guidelines (15).

A MeSH term search was performed using the following MeSH terms and boolean operators “AND” and “OR”: ((purpura schoenlein-henoch[MeSH Terms]) OR (IgA vasculitis[MeSH terms])) AND (posterior reversible encephalopathy syndrome[MeSH terms]) for papers published between January 1, 1970 and October 20, 2020, identifying 14 publications.

As IgAV has been formerly known as Henoch-Schoenlein Purpura, an additional free text search using Boolean operators and the terms (“posterior reversible encephalopathy syndrome” OR “posterior reversible leukoencephalopathy syndrome” AND “Henoch-Schoenlein purpura” OR “IgA vasculitis”) resulted in *n* = 275 records.

To identify additional cases before the introduction of PRES diagnosis in medical records in 1997, the free text search was repeated, including the terms “seizure” and “encephalopathy,” resulting in additional *n* = 36 records.

**Figure 1:**
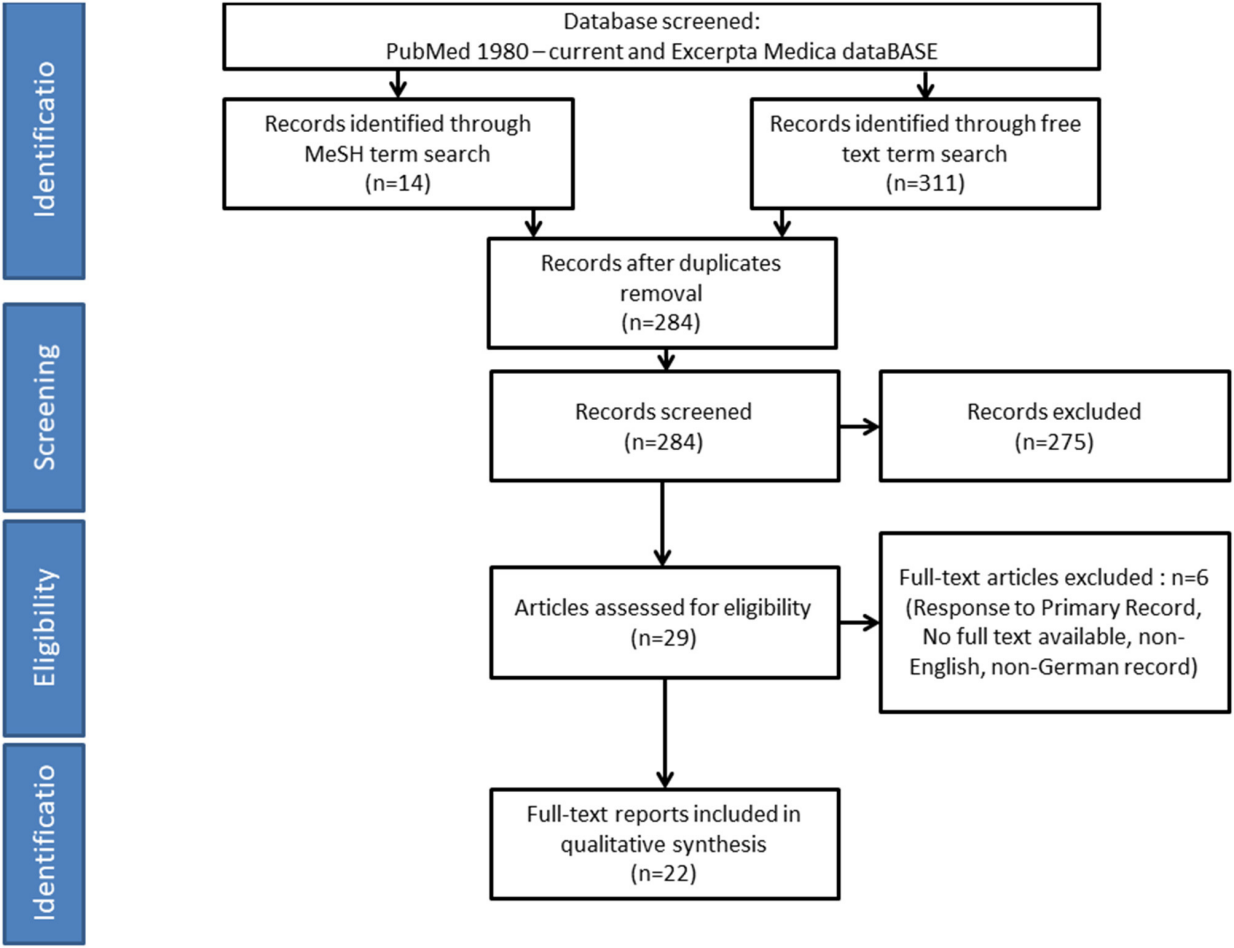
Literature review—summary of search and review process. Several reports included more than one patient, resulting in a total of 28 patients from 22 accessible reports.

We removed duplicates and considered for inclusion (systematic) reviews, original articles, case series, case reports, and conference papers with IgAV and encephalopathy or seizures in English or German (Figure 1).

Animal studies and human studies in which encephalopathy diagnosis or diagnostic criteria were not specifically reported were excluded.

Two independent researchers assessed studies for eligibility and quality, collected, and extracted data. Due to the heterogeneity of the data, only descriptive statistical analysis was performed.

## Results

### Clinical Report, Presentation, and Diagnostic Workup

A 7-year old previously healthy girl of non-consanguineous Caucasian parents was admitted due to the first occurrence of a brief self-limiting afebrile focal onset seizure (twitching of the right arm). Retrospectively, the patient reported intermittent abdominal pain and a brief period of intermittent double vision preceding the seizure by ~4–5 h on the day of admission.

Initial blood tests showed a normal hemogram, blood glucose levels (5.8–6.3 mmol/L), coagulation parameters, and renal retention parameters. CRP was mildly elevated to 22 mg/l [n.v. <8] and D-dimers were increased to 3.8 mg/l [n.v. <0.5].

**Figure 2:**
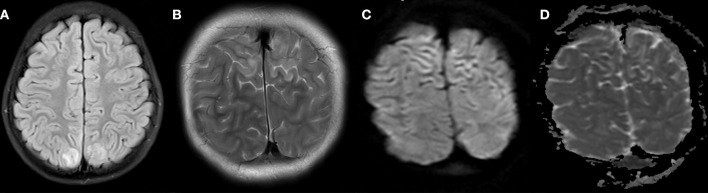
Neuroimaging: Axial T2-Fluid attenuated inversion recovery (FLAIR) **(A)**, coronal T2-weighted **(B)**, coronal diffusion-weighted images **(C)**, and with ADC map **(D)** from the initial MRI scan. Focal bilateral parietal cortical/subcortical high signal intensities with mild swelling were visible on T2-FLAIR and T2-weighted images **(A,B)**, on diffusion weighted images **(C)**, the affected areas showed largely no restriction, only vasogenic edema. Only small cortical sections were hypointense on ADC **(D)** indicative of cytotoxic edema.

Electroencephalogram (EEG) demonstrated severe bi-hemispheric slowing, especially in the right parieto-occipital region. Shortly after admission, the patient reported a sudden episode of abdominal pain, headache, loss of vision and suffered from another seizure with focal onset with evolution to generalized seizure activity. The seizure, together with the other symptoms, resolved after ~3 min without medical intervention. The cranial MRI (cMRI) performed on the first day of admission was consistent with PRES showing multiple spot-like, partly confluent cortical/subcortical hyperintensities predominantly in the bilateral parietal region on FLAIR/T2-weighed images with slight focal swelling (Figures 2A,B). On diffusion weighted images, the involved cortical ribbon and the adjacent subcortical white matter were hyperintense to a large extent without hypointensity on the ADC map indicating vasogenic edema. Only small cortical sections showed hypointensities on the ADC map in terms of diffusion restrictions. Typical watershed infarcts or microbleedings could not be detected. The patient's blood pressure was relatively low on the first night, with systolic pressures ranging from 98 to 100 mmHg and diastolic pressures ranging from 44 to 47 mmHg with a normal heartbeat rate of 86–125/min. Vasculitis workup was negative as demonstrated by normal von-Willebrand-Antigen, ANAs and p- und c-ANCAs, anti-ds ABs, ENAs, C3, C4. Cerebrospinal fluid showed no cells and normal levels of lactate, glucose, and protein. Microbiological workup and PCR investigations for neurotropic viruses were negative.

**Figure 3:**
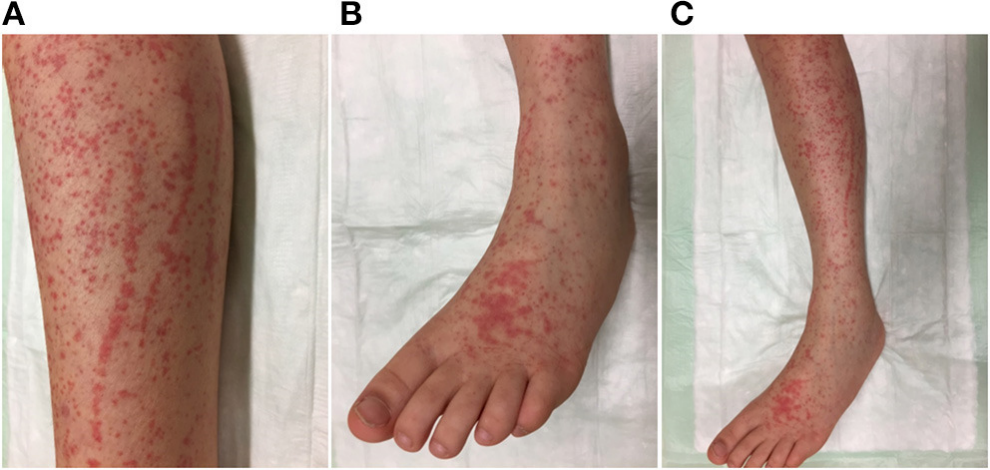
Skin lesions: Rash, appearing within the first 5 days after onset of the neurological impairment with hives, with small red spots or bumps on the buttocks, lower legs **(A,C)**, knees, feet **(B,C)**, elbows, and hands. The rash affected both sides of the body equally and did not turn pale on pressing. Occasionally, classical purpura was noted. The lesions completely resolved within 2 weeks.

Shortly after the admission, several petechiae developed on the forearms and lower limbs. Within the first 5 days of admission, the patient showed progressive petechial bleeding signs, which progressed to the classical and diagnostically groundbreaking palpable purpura (Figure 3), and suffered from another short episode of confusion and blurred vision. Concerning other organ manifestations, urine diagnostics and duplex-sonography of kidneys and gut demonstrated repeatedly normal findings. Arthralgia was not noted.

### The Course of the Disease, Treatment, and Follow-Up

After the second seizure on the day of admission, the girl suffered from headaches and mild behavioral changes but was overall in a stable condition. The patient was treated with acyclovir and ceftriaxone until a microbial etiology for the symptoms was excluded by blood- and spinal fluid analyses. In the course of the disease, blood pressure became elevated, reaching 150 mmHg systolic on the second day. Therefore, antihypertensive medication was initiated with dihydralazine and gradually extended with ramipril, amlodipine, and repeated doses of nifedipine, resulting in levels around 130/75 mmHg. Repeated abdominal ultrasound showed kidneys and gut normally perfused. Urine workup showed no hematuria, and an albumin/creatinine ratio of 33 mg/g ruled out renal impairment. Extended secondary hypertension diagnostics showed normal findings for ocular fundus, electrocardiogram, echocardiography, and endocrine disorders.

Four days after the seizure, a non-tender purpuric rash appeared on the lower extremity and buttocks/forearms/and on the trunk in loco typico for IgAV (Figure 2). Abdominal pain and nausea returned 2 days later, so intravenous methylprednisone 2 mg/kg/d was administered for 3 days. Daily abdominal ultrasound excluded intussusception. Under combined antihypertensive medication, the patient fully recovered neurologically without any further seizures. Blood pressure decreased to levels of 100 mmHg systolic under continued treatment with ramipril and amlodipine after 1 week.

The benefit of steroids in IgAV has only been shown for abdominal involvement (16), whereas no studies compare the neurologic outcome with steroids to no or other treatment. As our patient did not show neurological symptoms after the remission of the initial seizure during her entire stay and for the potential additional effects on the blood pressure, we did not administer steroids until the return of abdominal symptoms (4, 16). Following three courses of i.v. methylprednisone (2 mg/kg), which were well-tolerated, medication was switched to an oral equivalent and tapered. At 1-month follow-up, no neurologic residues were notable, EEG showed slow wave-complexes in the occipital areas and was otherwise normal, renal ultra-sound and urine workup remained without abnormalities. Hypertension diminished, and the antihypertensive medication could be discontinued after a total of 4 weeks. At 6-month follow-up, clinical and neurological examination and urine workup and blood pressure remained normal. EEG findings had significantly improved, showing only mildly slow waves in the occipital region. An externally acquired brain MRI follow-up was normal without residual lesions. Today, with a follow-up of 3 years, the patient remained symptom-free.

### Seizures and PRES in IgAV

Literature search with the aforementioned MeSH terms yielded 325 studies, of which 22 included patients with seizures or PRES associated with IgAV and two studies with IgAV associated with cerebral vasculitis. From these studies, clinical data of a total of 28 patients could be collected. Reports in English and German were evaluated independently by two authors. In addition, citations from reviews were checked, but no further reports could be identified by this (Table 1).

**Table 1:** List of all 28 and our novel patients suffering from IgAV and neurological impairment.

	**References**		**Patient details**				**Clinical symptoms**											**Neurologic involvement**			
	**Ref**	**Ref #**	**Sex**	**Age**	**[d] until start neuro**	**FU** **[month]**	**Purpura**	**Rash**	**Abd. Pain**	**Athralgia/** **-FU itis**	**Headache**	**Confusion**	**Loss/** **disturb. vision**	**Foc**. **seizure**	**Gen**. **seizure**	**Nephritis**	**Hypertonus**	**PRES** **diagnosis**	**Cer. vasc**.	**Remission**	**Neuro** **Sequelae**
1	Fidan et al., 2016	(10)	F	8	10	4		pr	pr							pr, ARF					
2	Sivrioglu et al., 2013	(17)	F	5	7	1	pr	pr	pr	pr	pr					ARF					
3	Sasayama et al., 2007	(18)	F	13	900			pr	pr							ARF					
4	Khokhar et al., 2016	(19)	M	22	regularily	1	pr	pr	pr		pr	pr			pr						
5	Stefek et al., 2015	(6)	M	8	10	12		pr	pr												
6	Arslan et al., 2018	(20)	M	14	7	2	pr	pr	pr	pr											
7	Pacheva et al., 2017	(7)	F	8	3	4			pr												
8	Özcakar et al., 2004	(21)	M	10	10	24		pr	pr												
9	Fuchigami et al., 2010	(22)	F	7	8	5	pr	pr	pr												
10	Current patient		F	8	7	6			pr		pr	pr									
11	Kim et al., 2014	(23)	F	8	7		pr	pr		pr											
12	Emeksiz et al., 2014	(9)	M	13	14																
13	Dasarathi et al., 2012	(24)	F	11	10	5			pr												
14	Ninomiya, 2019	(25)	M	8	4	2.5			pr										**RCVS**		
15	Shen et al., 2017	(26)	F	10	>32	8		pr	pr												
16	Belman et al., 1985	(27)	M	7	14	1			pr		pr										
17	Belman et al., 1985	(27)	F	7	8	1			pr												
18	Camacho et al., 2014	(28)	M	5	8	3			pr												
19	Palesse et al., 1989	(29)	M	13	>30	18				pr	pr					ARF					
20	Eun et al., 2003	(30)	M	8		4	pr		pr	pr											
21	Özkaya et al., 2007	(31)	F	10	18	1			pr												
22	Wen et al., 2005	(32)	F	13	25	1	pr	pr		pr								*enceph			
23	Woolfenden et al., 1998	(8)	M	10	5	1			pr												
24	Fösel et al., 1990	(33)	M	7	10	4.5			pr												
25	Lerkvaleekul et al., 2016	(34)	M	4	6		pr	pr	pr												
26	Perez et al., 2000	(35)	M	42	regularily	18	pr		pr	pr	pr							*enceph.			
27	Shen et al., 2017	(26)	F	6	12		pr	pr	pr							pr					
28	Shen et al., 2017	(26)	M	10	9	1			pr												
29	Bose et al., 2019	(36)	F	30	9	6	pr	pr	pr	pr						ARF					

Black, affected; white, not affected; gray, information missing. Pr, presenting sign; F, female, M, male, FU, follow up. Ref, Reference given as the name of first author and year of publication; Ref #, number of the cited publication in the reference list of this article. [d] until neuro, days from the presenting symptoms until the first clinical-neurological symptoms; and. pain, abdominal pain; loss/disturb. vision, visual disturbance as defined as disturbed vision or loss of vision; foc. seizure, focal seizure with or without secondary generalization; gen. seizure, generalized tonic-clonic seizures; nephritis, nephritis or other form of renal impairment up to ARF, acute renal failure; cer. vasc., confirmed diagnosis of cerebral vasculitis; RCVS, reversible cerebral vasoconstriction syndrome; *enceph, encephalopathic patients, reported diagnosis of cereb. Vasculitis but PRES possible; neuro sequelae, clinically manifest neurological impairment of body functions, imaging or EEG findings consistent with functionally not relevant scars after, e.g., infarction were counted as remission.

**Figure 4:**
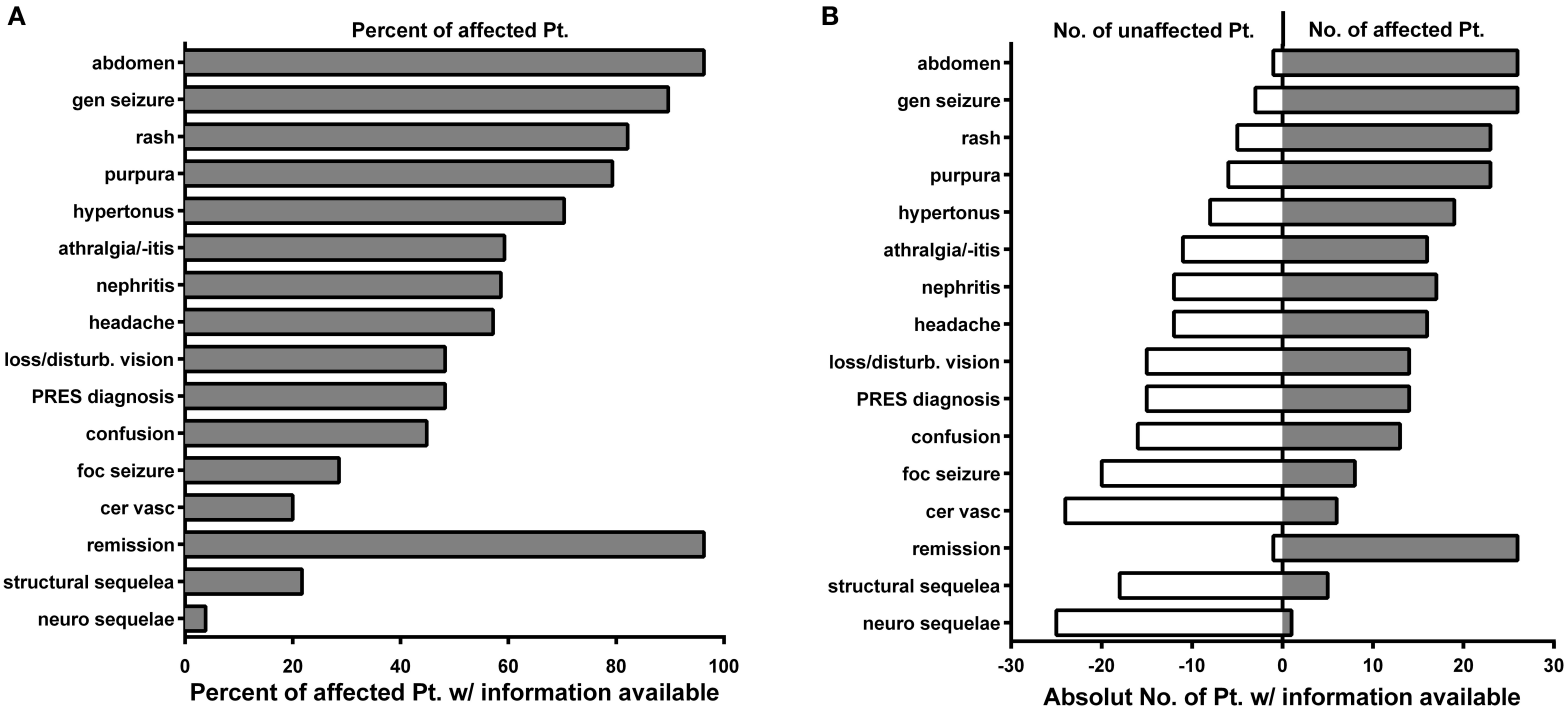
The clinical phenotype of IgAV-patients in this cohort **(A)** Fraction of affected patients relative to the whole cohort of patients of whom information was available with different clinical manifestations depicted on the Y-axis. **(B)** Absolute numbers of patients who are not affected by different clinical manifestations are depicted on the left (open white bars), while the absolute number of reported patients affected are depicted to the right side (solid gray bars). gen. seizure, generalized seizure; loss/disturb. vision, loss/disturbed vision; foc. Seizure, focal seizure; cer. vasc., cerebral vasculitis; neuro sequelae, neurological sequelae.

In total, we identified 28 cases with IgAV and reversible encephalopathy (6–10, 17–36). Twenty six cases were associated with seizures (6–10, 17–19, 21–31, 33, 34, 36), and thereof 14 cases fulfilled all clinico-radiological criteria of PRES (6, 7, 9, 10, 17–25). Lava et al. indicated the existence of seven other cases, which, however, were not available in English or did not fulfill the radiologic diagnostic criteria of PRES and were therefore not considered a confirmed PRES diagnosis in this summary (37). In contrast to the general prevalence of IgAV (male 2:1 female), neurological involvement in pediatric patients was nearly equally contributed, with 13 girls and 13 boys affected, respectively. Two cases of adult males (19, 35) (22 and 42 years of age) and one female patient were found (36). Bérubé et al., however, reported a 1.5:1 preponderance of male patients (38). The mean age of patients was 11.2 years (median 8.0, range 5-42 years), and no ethnic prevalence was found. The clinical spectrum of the given IgAV cohort is summarized in Figure 4.

Notably, abdominal pain was the most common presenting sign (26/30), similar to our patient's medical history. The majority (13/14) of patients fulfilling all diagnostic criteria of PRES (14/30), as defined by Fugate et al., experienced generalized seizures (13). From the cases with data available (28/30), neurological symptoms were noted on average 11.8 days after the first symptoms in pediatric patients (28/30). Both male adults suffering from reversible encephalopathy after IgAV experienced multiple episodes over several years after the initial diagnosis. Of note, the case of a 30-year-old Caucasian woman was the only reported case with a similar disease progression as seen in pediatric patients (36). However, the MRI of the patient's brain showed only non-specific foci of signal abnormality in the white matter and was therefore not indicative of PRES. Naturally, the most affected areas of the brain were occipital and parietal regions throughout imaging and EEG diagnostics.

While all patients with PRES showed at least mildly elevated blood pressure, only half of these patients (7/14) had renal impairment, ranging from nephritis to acute renal failure. Garzoni et al. (39) reported severe renal involvement as the most common (29 of 54 patients) concomitant feature in IgAV patients with neurological dysfunction (13). This indicates a close correlation between the severity of renal disease and neurological features. Interestingly, most cases of IgAV-associated encephalopathy showed full neurological remission (27/30), and thereof all 14 patients with PRES recovered fully. Of note, comparing our patient to the historical cohort, it becomes clear that she differed from previous reports in that seizures and PRES preceded the development of hypertension and purpura, respectively. From a patient perspective, the uncertainties of the diagnostic process due to the very unusual presentation in the initial phase of the disease were burdensome.

## Discussion

Although the long-term prognosis of IgAV is mostly attributable to renal impairment, cerebral involvement may produce substantial morbidity and mortality (13). Severe neurological symptoms have been reported in 2–8% of IgAV cases (27, 37, 40). More recent studies tend to report a lower incidence, e.g., Trapani et al., who report 3% with neurological involvement (40). These patients usually show good recovery within 94 and 89% of children and adults. Nevertheless, an estimated 20% of patients with IgAV who experience cerebral impairment suffer from long-term effects such as focal neurologic deficits or localization-related epilepsy (38, 41). Notably, half of those patients had developed intracranial hemorrhage.

In almost all patients with significant cerebral involvement included in this study, seizures occurred in the early phase of the disease. Only a fraction of the patients (8/29) suffer from focal, and 79.3% (23/29) had generalized convulsions (Table 1). These numbers are remarkably higher than the ~53% of all pediatric and adult IgAV patients, as reported by Bérubé et al. (38). However, only patients with the diagnosis of PRES were analyzed for this study. The majority (13/14) of PRES patients identified in this study suffered from seizures. This finding is in line with data (17/17 patients) reported by Lava et al. (37). The lower total number of patients in this study is due to the exclusion of patients in whom the ultimate diagnosis was ambiguous. PRES refers to a clinic-radiological disorder. However, there are no validated diagnostic criteria, and most literature is based on case-series or observational data (42). PRES has been increasingly recognized during the last two decades but still lacks prospective observational studies. Therefore, we opted to exclude older reports when lacking essential clinic-radiological data to diagnose PRES to prevent confusion in the field.

Interestingly, almost half of the overall IgAV patients (who had not suffered from seizures) showed abnormalities in EEG (43). This is in line with a recent EEG study reporting non-convulsive seizures and epileptiform patterns in 62% of the cases during continuous EEG monitoring of 32 patients with PRES (44).

In our patient, a brief afebrile focal spell (twitching of the right arm) that subsequently progressed to a generalized seizure with spontaneous resolution after 3 min led to hospitalization. Though seizures appear predominantly in the acute phase of the disease, we found three previously reported patients with seizures as presenting sign of IgAV in the literature (8, 24, 27). We conclude that IgAV should be considered a differential diagnosis of seizures, especially with signs of normo-thrombocytic petechia despite lack of CNS inflammation, and cMRI should be performed promptly.

PRES is usually characterized by headache, confusion, seizures, and visual loss as a result of focal vasogenic brain edema. PRES in IgAV is thought to occur due to either cerebral vasculitis under normotensive conditions and/or cerebral vessel autoregulation dysfunction (i.e., secondary to renal hypertension), which is mostly in line with the previously reported IgAV-PRES patients (45) (Table 1). In both scenarios, the blood-brain barrier is compromised, facilitating vasogenic edema (46).

Our case illustrates that PRES in IgAV can develop with only mildly elevated blood pressure or normotension. Normotensive PRES is, among other reasons, associated with a wide range of disease states like intake of immunosuppressive medications, especially calcineurin inhibitors, eclampsia, postpartum hemorrhage, systemic lupus erythematosus, and renal failure (12). Normotensive PRES in the context of IgAV has otherwise only been reported in three cases. In two of these cases, CSF and imaging findings were suggestive of inflammation and cerebral vasculitis. Interestingly, our patient initially suffered from (near) normotensive PRES without signs of cerebral vasculitis (Figure 3). In the recent case of an 8-year-old boy, PRES occurred with only mild hypertension and was complicated with reversible cerebral vasoconstriction syndrome (RCVS) (25). Cerebral vasoconstriction is occasionally seen in adult PRES patients. However, the highlighted case was the first presentation of both PRES and RCVS in a pediatric patient. In our patient, cMRI showed no signs of vasculitis or multifocal infarctions, as seen in the aforementioned patient. However, digital subtraction angiography has not been performed.

Other causes of PRES, such as trigger drugs or renal impairment, had been excluded. Park et al. suggest a role of interleukin-6 triggered vascular endothelial growth factor-induced angiogenic activity in the development of PRES after IgAV (47). However, IL-6 was only mildly elevated to 31 ng/l [n.v. <7] in the patient reported here, while cMRI showed perivascular edema. Therefore, we favor the hypothesis that the combination of possibly subclinical cerebral vasculitis impairing the blood-brain barrier in combination with mildly elevated blood pressure is sufficient to cause this severe clinical condition.

In IgAV, complexes of immunoglobulin A (IgA) and complement component 3 (C3) are deposited in the wall of arterioles, capillaries, and venules (48). Vessel wall friability and thrombogenicity of active vasculitis, antiphospholipid antibody synthesis, and other hemostatic disturbances may contribute to hemorrhagic and thrombotic complications of IgAV (37). We hypothesize that the inflammatory alteration of the vessel walls is most likely accompanied by dysfunction of autoregulation, resulting in elevated blood pressure (49). Due to limited sympathetic innervation of the posterior cerebral circulation, posterior regions are more vulnerable, and vasogenic edema is observed predominantly in those regions.

In the following days after the diagnosis of PRES had been made, our patient's blood pressure eventually rose markedly and was resistant to antihypertensive drugs. However, no additional neurological symptoms occurred during the following weeks of continuous and therapy-refractive hypertensive blood pressure.

Unlike PRES, hypertensive encephalopathy (HE) is defined as brain dysfunction due to significantly elevated blood pressure (50). Symptoms may include headache, vomiting, disequilibrium, confusion, and seizures—and, therefore, is quite similar to the clinical presentation of PRES. In hypertensive encephalopathy, the blood pressure is generally >200/130 mmHg, but occasionally it can occur at a BP as low as 160/100 mmHg. Hypertensive encephalopathy is most commonly encountered in young and middle-aged patients with uncontrolled hypertension. The mean systolic blood pressure reported in pediatric HE patients ranges from 175 mmHg in renal-origin hypertension to 137 mmHg in non-renal-hypertension (51). HE is a clinical condition caused by elevated blood pressure and which can, per definition, be reversed by blood pressure reduction. Untreated, however, it may also lead to the development of PRES, intracerebral hemorrhage, or ultimately death. Woolfenden et al. pointed out that earlier cases published as “cerebral vasculitis” had an evolution closely resembling PRES, which was first described in (8). Both conditions may especially affect the posterior brain regions. Diffusion-weighted imaging (DWI) and apparent diffusion coefficient (ADC) maps are used to characterize ischemic lesions of vasculitis and help to distinguish vasogenic edema. PRES lesions are typically FLAIR-positive, and only in severe cases, irreversible ischemia with pathological DWI and ADC signal occur. Inflammatory changes in the CSF may help to distinguish vasculitic from edematous lesions. However, both pathologies may involve normal CSF findings, and therefore, in fact, coexist. Bérubé et al. suggest that in the absence of hypertension or renal injury, direct vasculitic involvement of cerebral vessels may indeed play a direct causal role in PRES (38).

In summary, we conclude that severe CNS involvements such as seizures and PRES are underappreciated features of IgAV, which physicians should be made aware of. Furthermore, our case and three (8, 24, 27) previous reports indicate that CNS symptoms may precede other manifestations of IgAV. Thus, IgAV may initially mimic other conditions such as Waterhouse-Friderichsen syndrome. Lastly, the pathogenesis of PRES in the context of IgAV remains elusive. We show that PRES can occur despite the lack of renal hypertension or cerebral vasculitis, hampering educated treatment decisions. We speculate that hypertension in our patient, which developed after making the PRES diagnosis, was rather caused by PRES than causative for the PRES.

From a pediatric perspective, it is noteworthy that almost all patients (28/29) with neurologic manifestations presented with (25/27) or developed (2/27) diffuse to colicky abdominal pain. Similar cases have been described in young adults for the initial presentation of, e.g., acute intermittent porphyria or systemic lupus erythematosus complicated by PRES (52, 53). In all three entities, abdominal pain was one of the first signs of the systemic manifestation of the underlying disease. The combination of severe abdominal symptoms in young patients without medical history, inconclusive laboratory workup, and ultrasound should prompt the treating physician to consider an inflammatory disease with vascular involvement. Blood pressure and renal function should be monitored closely. Additional rash or mild neurological symptoms should be considered warning signs. Our patient presented with PRES and transient hypertension, leading to hospital admission. Increased awareness can help to direct the diagnostic workup and anamnesis of severe neurologic involvement and hypertension to vasculitic impairment, even before the appearance of the pathognomonic purpura. Timely neurological evaluation and neuroimaging, including diffusion-weighted sequences and cerebral angiography, can crucially contribute to prompt diagnosis and treatment.

## Data Availability Statement

The original contributions presented in the study are included in the article/supplementary material, further inquiries can be directed to the corresponding author/s.

## Ethics Statement

Written informed consent was obtained from the minor(s)' legal guardian/next of kin for the publication of any potentially identifiable images or data included in this article.

## Author Contributions

DF and CK conceived and designed the study, generated and analyzed data, and wrote the manuscript. FG, EB, and JG-A generated data and contributed to the manuscript. IH, FD, and UB contributed to the manuscript. CK supervised and coordinated the study. All authors contributed to the article and approved the submitted version.

## Conflict of Interest

The authors declare that the research was conducted in the absence of any commercial or financial relationships that could be construed as a potential conflict of interest.

## Publisher's Note

All claims expressed in this article are solely those of the authors and do not necessarily represent those of their affiliated organizations, or those of the publisher, the editors and the reviewers. Any product that may be evaluated in this article, or claim that may be made by its manufacturer, is not guaranteed or endorsed by the publisher.
